# In Silico Assessment of Safety and Efficacy of Screw Placement for Pediatric Image-Guided Otologic Surgery

**DOI:** 10.3389/fsurg.2021.736217

**Published:** 2021-09-29

**Authors:** Jan Hermann, Fabian Mueller, Stefan Weber, Marco Caversaccio, Gabriela O'Toole Bom Braga

**Affiliations:** ^1^ARTORG Center for Biomedical Engineering Research, Faculty of Medicine, University of Bern, Bern, Switzerland; ^2^Department of Otorhinolaryngology, Head and Neck Surgery, Inselspital, University Hospital Bern, Bern, Switzerland

**Keywords:** image-guided surgery, pediatric, cortical layer, screw placement, skull thickness, robotics

## Abstract

**Introduction:** Current high-accuracy image-guided systems for otologic surgery use fiducial screws for patient-to-image registration. Thus far, these systems have only been used in adults, and the safety and efficacy of the fiducial screw placement has not yet been investigated in the pediatric population.

**Materials and Methods:** In a retrospective study, CT image data of the temporal region from 11 subjects meeting inclusion criteria (8–48 months at the time of surgery) were selected, resulting in *n* = 20 sides. These datasets were investigated with respect to screw stability efficacy in terms of the cortical layer thickness, and safety in terms of the distance of potential fiducial screws to the dura mater or venous sinuses. All of these results are presented as distributions, thickness color maps, and with descriptive statistics. Seven regions within the temporal bone were analyzed individually. In addition, four fiducial screws per case with 4 mm thread-length were placed in an additively manufactured model according to the guidelines for robotic cochlear implantation surgery. For all these screws, the minimal distance to the dura mater or venous sinuses was measured, or if applicable how much they penetrated these structures.

**Results:** The cortical layer has been found to be mostly between 0.7–3.3 mm thick (from the 5^th^ to the 95^th^ percentile), while even thinner areas exist. The distance from the surface of the temporal bone to the dura mater or the venous sinuses varied considerably between the subjects and ranged mostly from 1.1–9.3 mm (from the 5^th^ to the 95^th^ percentile). From all 80 placed fiducial screws of 4 mm thread length in the pediatric subject younger than two years old, 22 touched or penetrated either the dura or the sigmoid sinus. The best regions for fiducial placement would be the mastoid area and along the petrous pyramid in terms of safety. In terms of efficacy, the parietal followed by the petrous pyramid, and retrosigmoid regions are most suited.

**Conclusion:** The current fiducial screws and the screw placement guidelines for adults are insufficiently safe or effective for pediatric patients.

## Introduction

Image guidance provides a technological solution for accurate anatomy and instrument localization through anatomy-to-image registration (1). Registration is the process of finding the transformation that maps a point in the anatomy with a corresponding point on the image. Commonly used methods include paired-point-based registration (with fiducial points), surface-based registration and more rarely automatic registration (2). Additionally, image-guided surgery (IGS) uses tracking systems which allows real-time determination of instrument position. The tracking system is based on either electromagnetic or optical tracking and both require the placement of an intraoperative tracker (2, 3). Lastly, the image is transferred to the IGS software and the displayed information can be used to localize in real time anatomical structures.

Otorhinolaryngological applications to date are mainly focused on rhinology. In endoscopic sinus surgery (ESS), the use of image guidance has demonstrated a potential to improve surgical outcomes (1). Achieving total resection in tumor cases, confirming complete anatomical dissection, and assisting in intraoperative decision-making are some of the applications of IGS. Recently, this technology has been applied in cochlear implant (CI) surgery with the use of fiducial screws for registration (3, 4). This requires the surgeon to implant and manually localize on the temporal bone each fiducial screw with a registration probe. The screws must be rigidly fixed to the skull and remain immovable on the bone. Failure to do so can result in registration error, inaccurate targeting, or damage to the related structures at the implantation and surgical sites (2, 5). The length and the number of the screws used vary according to the technology applied (e.g. template based, mechatronic arm). The Hannover group (6) uses five micro titanium screws of 1.5 × 6 mm (Martin, Tuttlingen, Germany) for registration, and a bone-anchored cranial reference array (BrainLAB AG, Munich, Germany), to reference the object and a reference adapter clamped to the surgical drill (Aesculap, Tuttlingen, Germany). Meanwhile, the template-based technique from Vanderbilt University uses 3 titanium anchors of 4 mm wide and 8 mm long with 4 mm of this length screwed into the skull (7). Currently, the Bern group (mechatronic arm) uses five 4 mm thread length titanium screws implanted on the skull, either bone screws intended for orthognathics (3, 8) or task-specific screws for image-guided application on the lateral skull base (9, 10). All these technologies are presently only used for adult patients.

Recent guidelines for cochlear implant surgery recommend implantation in pre-lingual phase as early as 6 months old in order to obtain optimal hearing outcomes (11). The bone growth process of the mastoid in small children has been shown to create additional surgical obstacles, especially during the first 4 years of life. Dura exposure during well preparation and facial nerve vulnerability at the mastoid tip are common in early childhood. In general, the posteroinferior portion of the temporalis muscle and along the supra-mastoid crest bear a bone thickness between 3–6 mm depending on age (10, 12, 13). Additionally, the growth process of the mastoid bone during childhood is accompanied by the increase in mastoid pneumatization. This ceases around puberty, with the development of the last air cells in the petrous apex (14). These air cells can create further difficulty for screw placement for it decreases the thickness of the cortical layer of the bone, which is defined as the dense outer surface of bone that forms a protective layer around the internal cavity (15). If the screw is placed in an air-cell, then screw stability, accuracy and procedure safety can potentially be compromised.

Another obstacle posed by the most recently described IGS techniques that can be applied in pediatrics for CI surgery is the need of a dynamic reference screw placement to bear the tracker as described in previous studies (3, 8, 10). This screw is usually placed towards the occipital region of the calvarium, increasing the chances of dura and vascular trauma. Recent studies for the use of IGS in pediatric subjects in CI surgery have demonstrated not enough bone support in some areas of the temporal bone, raising the need for age-specific screw designs (10). However, in the future it might not be necessary anymore to place multiple fiducial screws. Studies are being conducted to design a patient tracker that is fixed to the skull with only one screw and multiple legs, containing the necessary fiducials for IGS.

Hence, the primary aim of this study is to investigate the efficacy and safety of screw placement in pediatric cases. The efficacy is measured in terms of the thickness of the cortical layer of the temporal bone (CLT – cortical layer thickness) derived from computed tomography data of pediatric cases. The safety is measured in terms of distance to dura mater or venous sinuses (DDVS – distance to dura or venous sinuses), i.e. sigmoid and transverse sinus. The secondary aim is to further evaluate the safety of screw placement by implanting screws in patient-specific 3D-printed models, and subsequently investigating their distance to the dura and the venous sinus (4 mm thread length screws).

## Materials and Methods

### Image Data

With permission of the local institutional review board (KEK 2017-01722), CT image data from 11 subjects meeting inclusion criteria (8–48 months at the time of surgery, CI surgery at the Otolaryngology Department of Inselspital between 2014 and 2017) were selected, resulting in *n* = 20 sides included in the study protocol. The two remaining sides were discarded due to image quality issues. An algorithm was used to estimate the thickness of the cortical bone and the distance of the fiducial screws to anatomical structures (dura and venous sinuses).

### Phantom Preparation

**Figure 1:**
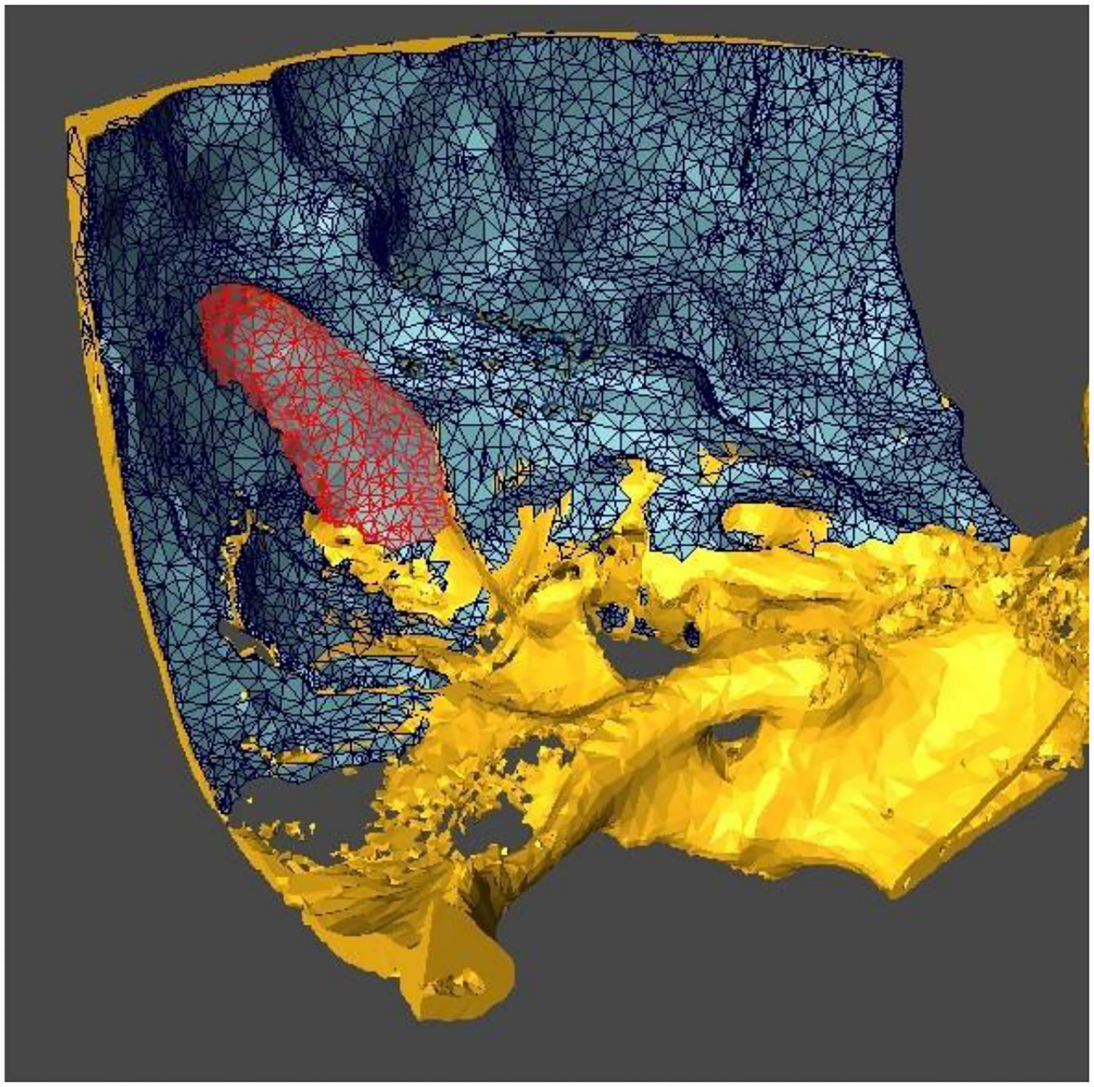
Segmented temporal bone (yellow), dura (blue and black lines) and sigmoid sinus (red).

Using medical image analysis software (Amira, Thermo Fisher Scientific, Waltham, Massachusetts, USA) the temporal bone, dura and sigmoid sinus were segmented (Figure 1). Surface models of the temporal bone were used for stereolithographic 3D-printed phantom creation (Eden260VS, Stratasys, MN, USA) where the fiducial screws were implanted following manufacturer's guidelines.

**Figure 2:**
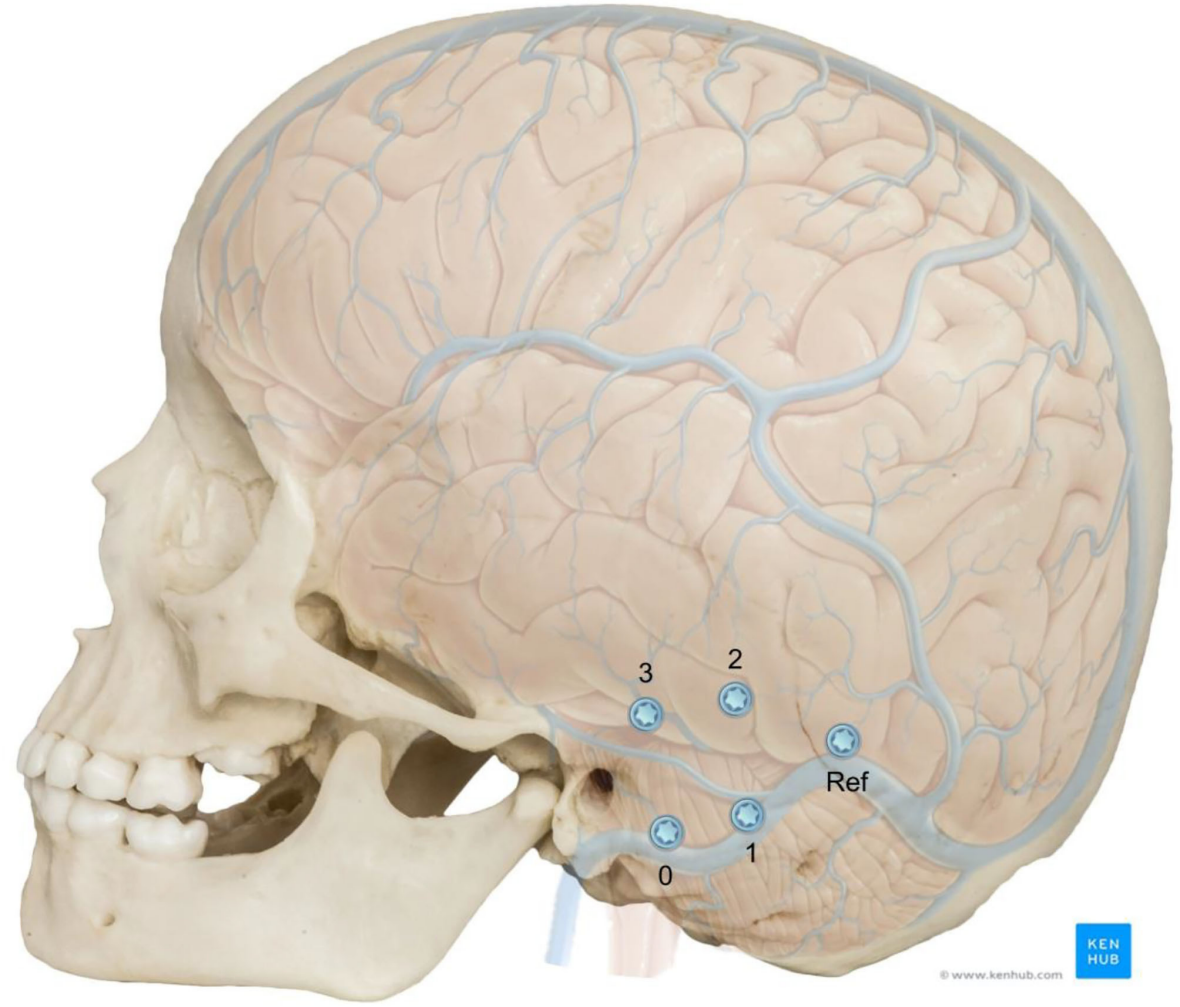
Adjusted position of the fiducial screws relative to the neurovascular anatomy in a 5 year old skull. The screws positions were numbered for better identification and the reference screw position is shown. Image adapted from sources: © Bone Clones (www.boneclones.com); © Kenhub (https://www.kenhub.com/en/library/anatomy/veins-of-the-brain), Illustrator: P. Kim.

Each of the phantoms were implanted with four titanium reference cone screws (3.2 mm thread diameter × 4 mm thread length, and 9.3 mm total length, CASCINATION AG, Bern, Switzerland) on the mastoid tip, posterior to MacEwen's triangle (temporal line superiorly tangent to external auditory canal and postero-inferior rim of the canal) and parallel to the temporal line and superior and posterior to the spine of Henle (Figure 2). The fifth screw, which is usually used to attach the patient tracker, was not placed in these phantoms. After insertion of the screws, high resolution computed tomography (CT) images (voxel size 0.15 × 0.15 × 0.2 mm^3^, XCAT XL, Xoran, MI, USA) were acquired. By means of registration through a mutual information approach (Amira), the original CT was co-registered to the phantom cone-beam CT scan (CBCT) and the relative positions of the screws were added to the segmented temporal bone anatomy.

### Thickness Analysis in Virtual Surface Meshes

A study was conducted to investigate the suitability of cortical bone thickness in the mastoid region at the potential sites for screw implantation. Available datasets were divided into four groups of different ages (group G1: 8–10 months old; group G2: 13–14 months old; group G3: 24 months old; group G4: 48 months old). Between all the aforementioned IGS techniques for CI surgery, we chose to use the most recent guidelines for screw placement provided by a commercially available otological surgical system (HEARO, CASCINATION AG, Bern, Switzerland). These techniques require the placement of five 4 mm thread length fiducial screws around the mastoid area.

**Figure 3:**
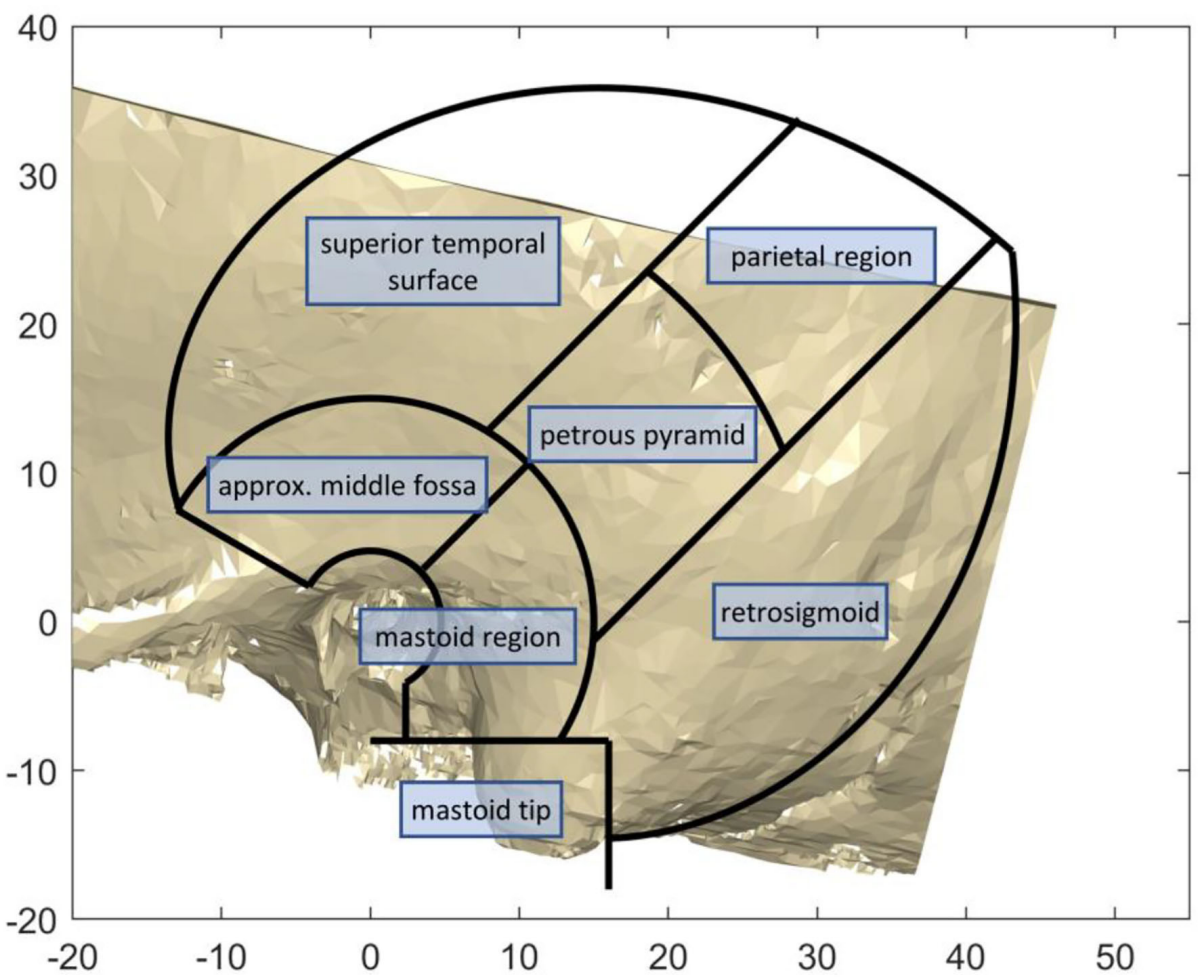
The anatomical regions of the temporal bone used for the analysis.

To investigate bone thickness, measurements were conducted in digital reconstructed models of the temporal bone, and the results analyzed statistically, as well as presented as thickness maps over the region of interest. Similar measurement methodologies in digital reconstructed models have been used in the literature (13, 16, 17). Temporal bone segmentation was achieved using threshold adjustments such that the air cells are included in the model, highlighting the cortical layer. All the temporal bones were aligned to a global axis to allow for calculation of descriptive statistics such as average measurements in the medical image analysis software Amira. The z-axis was chosen to be the auditory canal, pointing outwards. The x-axis is parallel to the temporal line pointing posteriorly. The region of interest for screw placement was described, as seen in Figure 3, first as a region between an angle of −60 and 30 degrees around the z-axis, with respect to the x-axis, and secondly between a radius of 15 and 50 mm from the origin in the external auditory canal with a linear angular relationship. The maximal distance of 50 mm lies along a line with an inclination of 30 degrees. The individual regions are separated as shown in Figure 3. The mastoid region is contained within a circle band of 15 to 30 mm in diameter, separated by a 45 degree line. The petrous pyramid is contained within a region at an angle of 45 degrees, with a band thickness of 15 mm, separated from the parietal region at a distance from origin at the center of the auditory canal of 30 mm. This region is shifted 4 mm in the negative y-axis direction. The mastoid tip region is defined as all space within the region of interest below 8 mm from the origin in the negative y-axis direction, and not further than 16 mm from the origin in the negative x-axis direction.

The dura mater and sigmoid sinus segmentation used the already segmented temporal bone, in a way that only the medial (interior) surface was segmented. The interior surface of the bone also contains channels from nerves and vessels, which were removed manually from the three-dimensional mesh. The fiducial screws were also segmented, and the axes were manually determined using the tip and screw head. All the data was exported as STL files providing a 3D representation.

The available cortical bone thickness in the temporal bone region, the CLT, was measured in the surface mesh data from the segmented temporal bones with a custom-developed script in MATLAB (MathWorks, Natick MA, USA). Thickness was calculated as the minimal distances from grid points projected onto the surface of the temporal bone to the nearest opposite surface. These opposite surfaces can be either an air cell or the interior surface of the temporal bone.

The distance from the surface of the temporal bone to either dura mater or the venous sinuses, the DDVS, was calculated as the minimal distances from projected grid points onto the surface of the temporal bone to a manually segmented dura and sigmoid sinus surface mesh.

The measurements of the distance of the fiducial tip to the dura or sigmoid sinus were carried out by first determining the screws head and tip points. Then, either the minimal distance was measured from the screw tips to the dura mesh, or how much the tip lies beyond the dura mesh along the axis of the screw.

## Results

**Figure 4:**
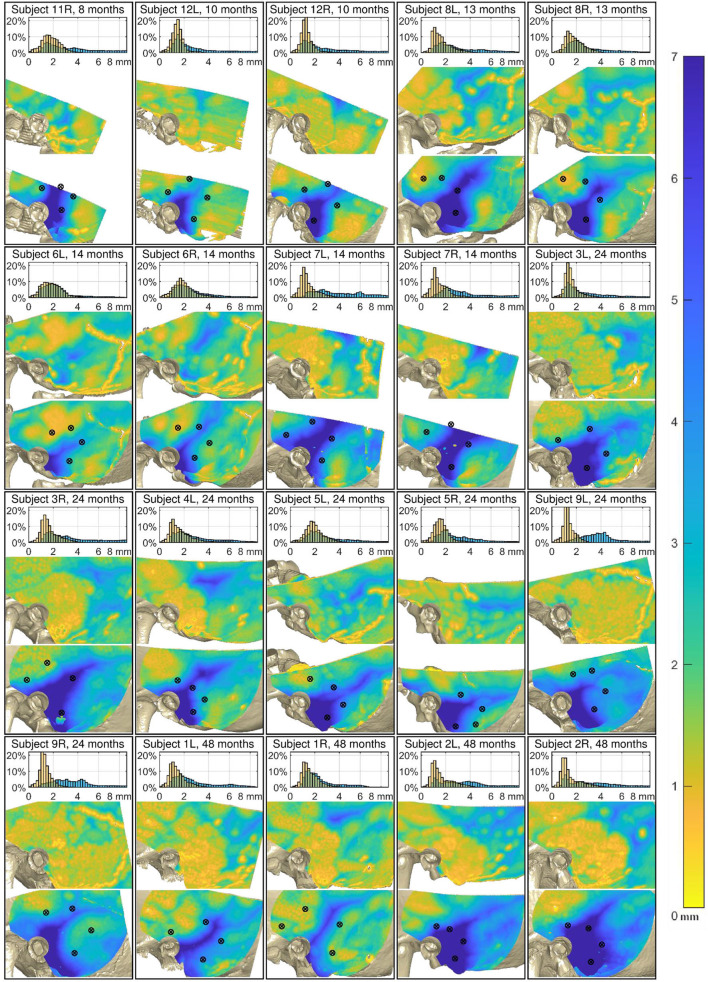
Analysis of the 20 cases. Top of each case: Relative distribution of the cortical layer thickness (CLT, orange) and the distance to the dura or venous sinuses (DDVS, light blue). Middle: The thickness of the cortical layer. Bottom: The distance to the dura or the venous sinuses. The black markers designate the locations where the fiducial screws were placed. The maps of all subjects are aligned relative to the external auditory canal and temporal line.

Without prior knowledge the screws can easily be placed in areas where the distance to the dura or the venous sinuses is short (Figure 4). In fact, the distance between the screws and the anatomical structures underneath expose a separation of less than 0 mm demonstrating potential trauma to the dura mater or the venous sinuses in all four age groups. From all 80 placed fiducial screws in the 3d-printed temporal bones, 22 would likely have touched or penetrated either the dura mater or the venous sinuses if it had been a patient.

The results show a great anatomical variance between the 11 subjects, even unrelated to age. A result overview is displayed in Figure 4, showing the histogram of the CLT and the DDVS. Additionally, both of these measurements are presented as color maps overlying the 3D reconstructed model from the CT scan. On these maps, the color corresponds to the measurement value, as indicated by the color bar on the right of the figure. The DDVS varied considerably between the subjects and ranged mostly from 1.1 to 9.3 mm on average (from the 5^th^ to the 95^th^ percentile), depending on the location.

**Figure 5:**
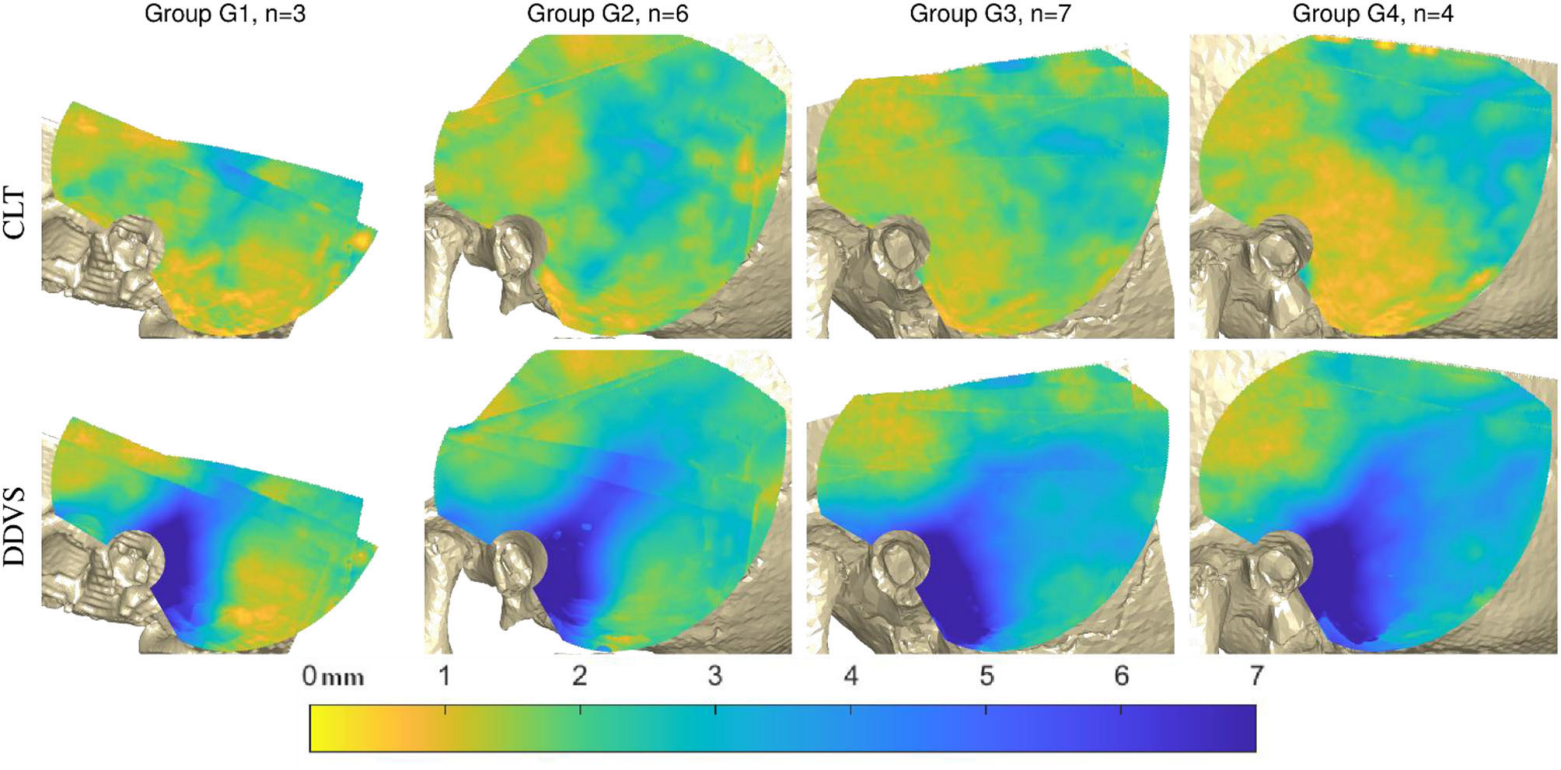
Analysis of the 20 cases in age groups. Top: the average cortical layer thickness (CLT). Bottom: the average distance to dura mater or venous sinuses (DDVS) in all subjects according to the age groups G1 (8–10 months old), G2 (12–14 months old), G3 (24 months old), and G4 (48 months old).

Figure 5 shows the average measurements taken on the reconstructed CT scan per subject groups based on age. While the mastoid pneumatization with air cells is not yet visible in the younger age groups G1, and G2, it can clearly be distinguished in groups G3, and G4.

**Figure 6:**
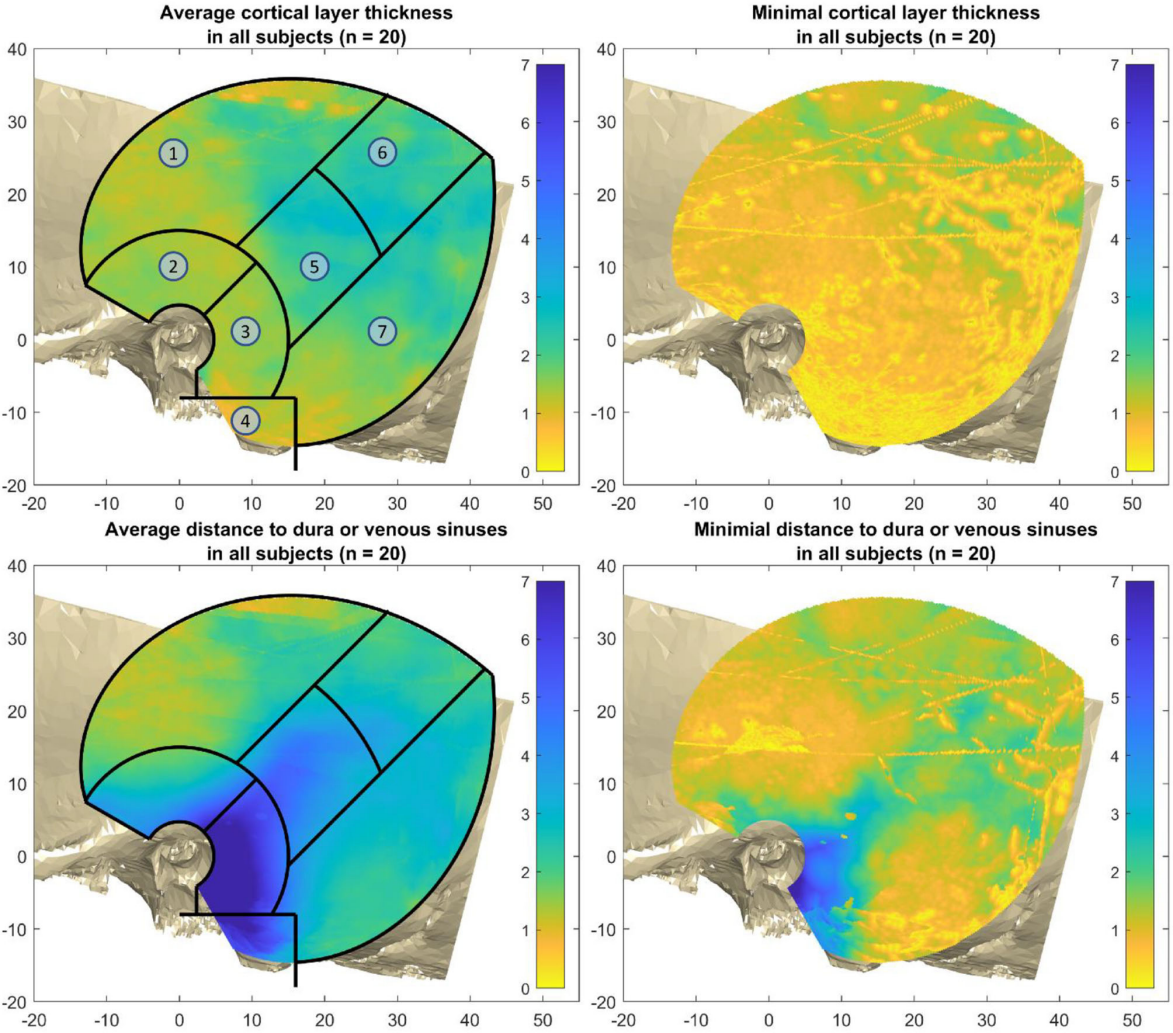
Average and minimal thicknesses over all 20 subjects. Top left: the average cortical layer thickness (CLT). Top right: the minimal cortical layer thickness over all subjects. Bottom right: The average distance to dura or venous sinuses (DDVS). Bottom right: the minimal distance to dura or venous sinuses over all subjects. The regions are as follows: 1) superior temporal surface, 2) approximately the middle fossa, 3) mastoid region, 4) mastoid tip, 5) petrous pyramid, 6) parietal region, and 7) retrosigmoid region.

The mastoid region and petrous pyramid can be clearly seen due to the long distance to the dura and venous sinuses (Figure 6, bottom left), while the superior temporal surface shows short distances (Figure 6, top left). The minimal cortical layer map show the minimal cortical thickness values encountered across all subjects. This map demonstrates how almost everywhere in the region of interest for screw placement, an air cell or thin cortical layer could be encountered, especially in the mastoid area. The fine yellow lines in that figure stem from either the borders of the reconstructed temporal bone models, or from the cranial suture lines (Figure 6, top right). The minimal distance map of the DDVS on the bottom right shows that in the mastoid region, along the petrous pyramid, and at small areas within the parietal and retrosigmoid regions, there are places where the minimal distance to the delicate anatomical structures dura mater and venous sinuses is greater or equal than 2 mm. However, this area is small and non-uniform (Figure 6, bottom right).

**Figure 7:**
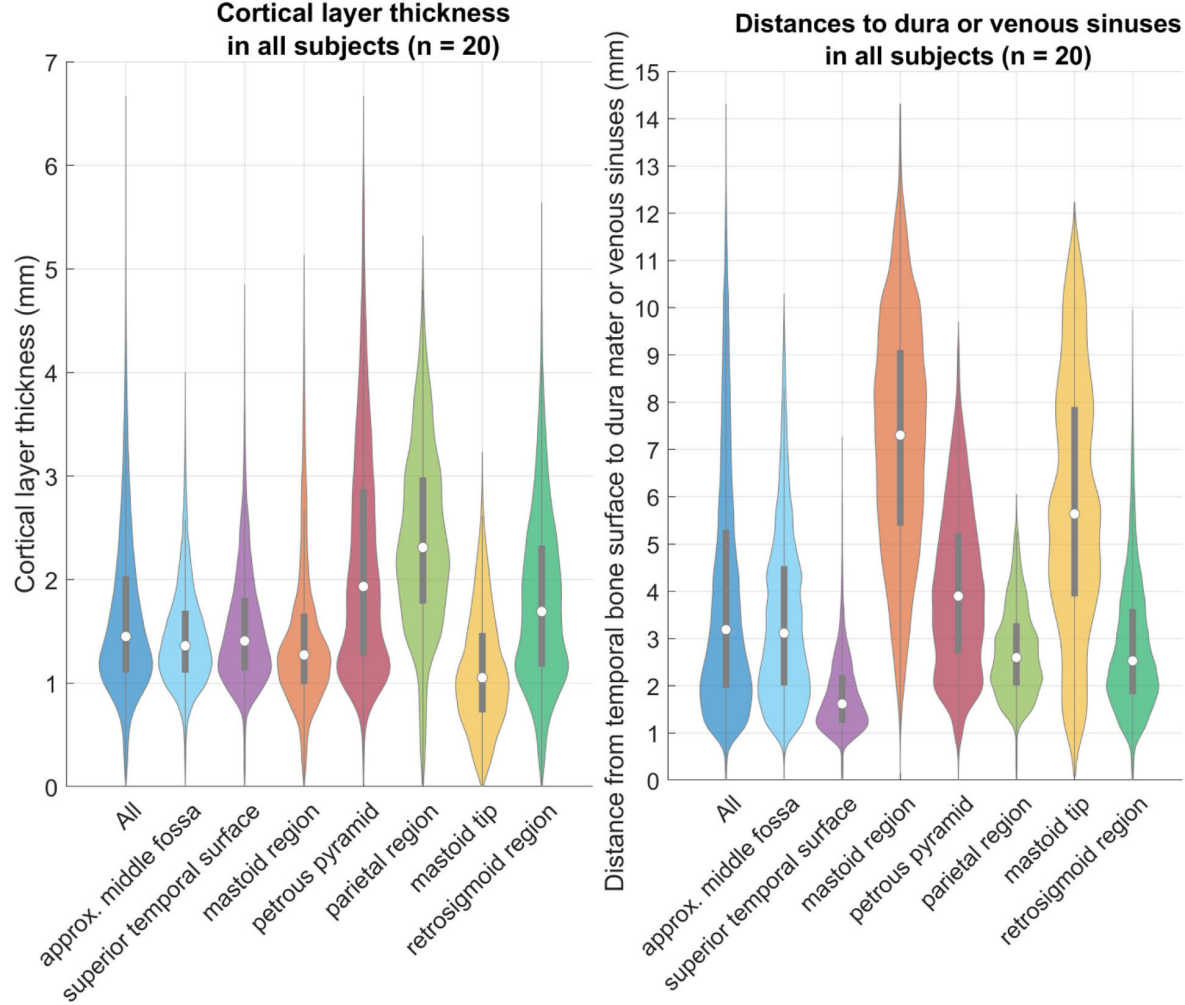
Left: relative distributions of the cortical layer thickness (CLT) in the individual regions as defined above. Right: relative distributions of the distance to the dura or venous sinuses (DDVS). These violin plots are a variation of boxplots, that also show the distribution similar to a histogram (18). The gray bars within the violin plots are the lower and upper quartiles, and the white circle is the median value.

The cortical layer has been found to be mostly 0.7–3.3 mm thick (from the 5^th^ to the 95^th^ percentile) in the region of interest for screw placement in all four age groups. In all age groups there are areas that are thinner than 0.7 mm, and areas that are thicker than 3.3 mm (Figure 7). For screw placement efficacy in terms of the cortical layer thickness, the parietal region followed by the petrous pyramid regions and retrosigmoid regions seem best, with cortical layer thicknesses of mostly 1.0–3.9 mm and a median of 2.3 mm, mostly 0.9–4.3 mm with a median of 1.9 mm, and 0.7–3.3 mm and a median of 1.7 mm, respectively. For screw placement safety in terms of the distance to dura or venous sinuses, the mastoid region is best with distances mostly between 2.0 and 11.1 mm and a median of 7.3 mm, followed by the petrous pyramid region with distances of 1.7–7.1 mm with a median of 3.9 mm (see Table 1, Figure 7).

**Table 1:** Select percentiles of both cortical layer thickness, and distance to dura mater or venous sinuses.

**Percentile**	**1^**st**^**	**5^**th**^**	**50^**th**^**	**95^**th**^**	**1^**st**^**	**5^**th**^**	**50^**th**^**	**95^**th**^**
**Region**	**CLT - cortical layer thickness (mm)**				**DDVS - distance to dura mater or venous sinuses (mm)**			
**All**	0.3	0.7	1.5	3.3	0.8	1.1	3.2	9.3
**Approx. middle fossa**	0.5	0.8	1.4	2.4	0.9	1.2	3.1	7.0
**Superior temporal surface**	0.5	0.8	1.4	2.6	0.5	0.9	1.6	3.3
**Mastoid region**	0.2	0.6	1.3	2.9	2.0	3.1	7.3	11.1
**Petrous pyramid**	0.6	0.9	1.9	4.3	1.0	1.7	3.9	7.1
**Parietal region**	0.4	1.0	2.3	3.9	1.0	1.4	2.6	4.5
**Mastoid tip**	0.1	0.3	1.2	2.2	0.9	1.5	5.6	10.3
**Retrosigmoid region**	0.3	0.7	1.7	3.3	0.7	1.1	2.5	5.5

The results of the cortical layer thickness and the distance to dura mater or venous sinuses are summarized in Table 1, containing the 1^st^, 5^th^, and 95^th^ percentile values, as well as the median over all 20 temporal bones.

## Discussion

Bone is a living tissue that is always under remodeling through a balanced process of resorption and formation that keeps bone integrity and homeostasis. Usually during the growth process the mastoid thickness increases from a minimum of 17 to 34 mm from 6 months to 20 years (19). These numbers are under the influence of factors such Eustachian tube permeability (that allows for middle ear ventilation through positive pressure), infection history, genetics and mechanical influences (20). Creating more or less aerated mastoids impacts on the thickness of the cortical layer and screw fixation. For example, case 5 (group G3, 24 months old) where the patient had bilateral ventilation tubes presenting a more aerated mastoid on the left side than the right side. Leading to a thicker cortical layer on the right side than on the left. Meanwhile, case 9 from the same age group but without evident history of middle ear disease, the patient presents less aeration than expected on both mastoids and thinner cortical layer. Other example are cases 3 and 4 (also in G3), where the mastoids have the expected aeration, a thin cortical layer with a thickness of no more than 2 mm is found. Similar situation is observed on cases 6 and 7 (in G2, 14 months old). Patient 6 presented soft density material taking over the mastoid and middle ear regions and a thicker cortical layer, typical of a diseased mastoid. While patient 7 had both cavities filled with air and a thinner cortical layer and, therefore, a higher chance of screw implantation in an air cell. Therefore, these cases demonstrate that not only the patient age should be taken under consideration but also their pathological history pose as a challenge for the use of IGS technology.

Due to the anatomical situation on the pediatric calvarium, the manufacturer's guidelines for screw positioning in adults had to be adapted to children. The mastoid tip actually has the lowest median cortical layer thickness, and a high median safety distance. However, in children the mastoid tip cannot be used as a screw placement due to the anatomical position of the facial nerve that is exposed until 2 years of age. This way, the screw placement had to be shifted to a more superior position, where the skull density (cartilaginous parts and not fully-ossified bones) create an additional obstacle. Causing the more superior screw to be often localized in the middle fossa region. Furthermore, to keep accuracy, a certain distance between the screws must be respected. Creating a smaller area for middle ear access trajectory placement in IGS surgery, while obeying the limits posed by the facial recess and cochlear angles on this age group. Although the mastoid bone itself present adequate thickness for the screws (10), the surrounding temporal bone is still not fully formed, so care must be taken to preserve enclosing anatomy. Especially the dura, sigmoid and transverse sinuses are at risk. Measurements of the positions of these structures are usually done with the aid of computed tomography, but excessive exposure to children to ionizing radiation should be avoided. The use of point-of-care ultrasound (POCUS) have also been mentioned in literature (21, 22) for fracture determination and bone-anchored hearing aid placement with promising results. The use of this technology can assist in screw placement for pediatric subjects before screw implantation.

The results show thin cortical layers in this pediatric population, which are mostly 0.6–3.2 mm thick (from the 5^th^ to the 95^th^ percentile) in the region of interest for screw placement across all subjects. Even if only the thickest regions (i.e. parietal, petrous pyramid, retrosigmoid) are considered, the thickness values are still in a similar range. Hence, it is likely that a thin cortical layer is encountered during screw placement, and the screws could become loose during surgery, leading to navigational errors and potentially dangerous situations. The results of the distance to dura mater or the venous sinuses are mostly 1.1–9.3 mm with a median of 3.3 mm. Thus, the screw placement with the thread lengths of the current IGS systems (i.e. around 4 mm) run into the risk of penetrating the dura mater or venous sinuses.

If a new screw thread length for this pediatric population had to be chosen, it would need to be only 0.8 mm long to be able to place the screw in 99% of the area of interest for screw placement across (see Table 1), and 1.1 mm long for 95% of the area of interest. If only the mastoid region, and petrous pyramid region was chosen, then the maximal screw thread length to place the screws in 99% of the area in the studied population (i.e. younger than 48 months old) would be 1.0, and 1.7 mm for 95%. Screws that are this small are bound to be less stable than the current screws, and thus could be dislocated easier. It would seem logical to adapt the registration probe to be as lightweight as possible, since in some systems currently the registration probe is a fairly heavy drilling end-effector with a registration tool inserted. The same logic applies to the patient tracker attached with the reference screw.

Limitations of this study are the manual rigid alignment of the anatomies for the statistics, and the small sample size. Calculating the average over multiple thickness maps includes the assumption that all data shares the same coordinate system. While the temporal bones have been rigidly aligned manually, no non-linear morphing of the anatomy was executed, which would make landmarks match locations (e.g. all ear canals, all mastoid tips, all temporal lines would be at the same coordinates). Especially in the data for the cortical layer, there are zero thickness values. These values stem mainly from the cranial suture lines, where the cortical layer thickness can be understood to be zero on the map. Although our sample size is small, some clinical considerations can be drawn regarding expected complications. Meningitis, fistula, thrombophlebitis, subdural empyema, otogenic suppurative thrombophlebitis, brain abscess and CSF (cerebral spinal fluid) leakage can be expected from damage to the dura. Venous air embolism, thrombosis, infarction, thrombophlebitis and death can happen if the sigmoid or the transverse sinuses (at risk with the reference screw positioning) are damaged. Although these complications are rare in daily CI surgical procedures, but with the introduction of new technology and its new requirements, care must be taken to avoid them.

This study demonstrated potential damage to the dura and venous sinuses in all screw positions for the pediatric population with the currently used fiducial screws and screw placement guidelines. The measurements taken show thin cortical layers, and short distances from the temporal bone surface to the delicate anatomy underneath.

## Conclusion

Due to the thin cortical layer and distance to vital anatomical structures (e.g. dura mater, sigmoid and transverse sinuses), the current fiducial screws and the screw placement guidelines might pose a challenge for safety and efficacy in image-guided surgery for patients younger than 48 months old. Therefore, an adaptation of current fiducial screws and/or their placement is necessary. Additionally, the use of image-guided technology (e.g. with navigated surface matching), or technologies such as ultrasound for screw placement could potentially assist in increasing safety and efficacy of the procedure.

## Data Availability Statement

The raw data supporting the conclusions of this article will be made available by the authors, without undue reservation.

## Author Contributions

GO'T and JH created the study design. SW and MC reviewed the study design. MC provided the data used in the study. JH and FM developed the software for the analysis of the virtual surface meshes of the anatomy. GO'T, FM, and JH conducted the phantom study on the additively manufactured models. GO'T and JH analyzed the collected data and wrote the manuscript. All authors reviewed the manuscript and approved the submitted version.

## Funding

This work was supported by the Swiss National Science Foundation SNF (Project 176007).

## Conflict of Interest

SW is cofounder, shareholder, and chief executive officer of CASCINATION AG, Bern, Switzerland, that commercializes the robotic cochlear implantation technology. The remaining authors declare that the research was conducted in the absence of any commercial or financial relationships that could be construed as a potential conflict of interest.

## Publisher's Note

All claims expressed in this article are solely those of the authors and do not necessarily represent those of their affiliated organizations, or those of the publisher, the editors and the reviewers. Any product that may be evaluated in this article, or claim that may be made by its manufacturer, is not guaranteed or endorsed by the publisher.
